# Handling Features of Patient Safety Incident Reporting Software and Shortcomings in Report Processing From Healthcare Professionals' Perspectives: A Cross-Sectional Study With a Qualitative Design

**DOI:** 10.1155/jonm/6724890

**Published:** 2025-10-02

**Authors:** Saija Koskiniemi, Tiina Syyrilä, Katri Hämeen-Anttila, Marja Härkänen

**Affiliations:** ^1^Department of Nursing Science, University of Eastern Finland, Kuopio, Finland; ^2^School of Pharmacy, University of Eastern Finland, Kuopio, Finland; ^3^Research Centre for Nursing Science and Social and Health Management, Wellbeing Services County of North Savo, Kuopio, Finland

## Abstract

**Background:**

Patient safety incidents are underreported, and report handlers, usually unit managers, are dissatisfied with the incident reporting software's handling features.

**Aim:**

To (1) identify the handling features of patient safety incident reporting software that support and challenge report processing; (2) determine which features report handlers believe should be added and (3) describe processing shortcomings from reporters' perspectives.

**Design:**

A cross-sectional study with a descriptive qualitative design.

**Methods:**

A descriptive qualitative cross-sectional study was conducted in two Finnish wellbeing service counties between January and February 2024. A total of 755 participants who used patient safety incident reporting software completed the Users' Perceptions of Patient Safety Incident Reporting Software survey. Their free-text responses (*n* = 117) were analysed using qualitative inductive content analysis.

**Results:**

Most respondents (66%) had a nursing background, and over half (51%) handled reports. Respondents had varying perceptions of software handling features that supported or challenged report processing, and they identified more features as challenging than supportive. They suggested changes to the anonymity and visibility of reports and the technical handiness of software. Respondents described the following report processing shortcomings: reports were not discussed within workplaces; discussion caused blaming; no concrete changes occurred after reporting; reporters did not hear about reports after reporting; reported incidents were underestimated and processing was not objective.

**Conclusion:**

The findings indicate that it is time to critically evaluate the usefulness of reporting software. Incident report handlers need optimum tools to process valuable client and patient safety information. Furthermore, incident report processing procedures require changes to assure reporters that it is meaningful and secure to report all patient safety incidents they observe or are involved in.

**Implications for Nursing Management:**

This study highlights the need for improvements in incident reporting software from the perspective of report processing. Additionally, report processing structures and methods must be clarified.

## 1. Introduction

Patient safety incident reporting systems are a crucial component for improving patient and client safety. However, approximately 1 in 30 patients remains exposed to preventable medication errors across healthcare settings [1], and millions of incidents have been reported worldwide after the implementation of electronic incident reporting systems [2]. Incident reporting systems have not reached their full potential [2]. The effect of systems on client and patient safety has been questioned [3]. While incident reporting systems gather information on incidents, their actual impact on practices is often minor [3]. One factor preventing reporting software from reaching its full potential is the challenge of taking sufficient actions in response to reported incidents [2]. In a previous study, after report processing, only 3% of reported incidents included written recommendations on how to avoid incident recurrence [4].

Incident reporting systems collect both structured and free-text patient safety incident data [5]. This study uses the term “reporting software” to refer specifically to electronic incident reporting systems. While some countries, like the United Kingdom, use national reporting software [3], others, like Finland, have multiple reporting software [6]. Nurses, the largest occupational group in healthcare globally [7], are the most often reporters of patient safety incidents [8], and many report handlers have a nursing background.

Patient safety culture is closely linked to incident reporting. Its dimensions consist of shared values, beliefs and behaviour norms related to patient safety [9]. Safety culture plays a central role in determining how reporting systems are utilised. An open, nonpunitive culture encourages reporting across continents [10], whereas punitive reporting processes lead to a reluctance to report [11]. Patient safety culture scores on the Safety Attitude Questionnaire among nurses, physicians and other health professionals were positively correlated with incident reporting and negatively correlated with incident occurrence in a study conducted in inpatient units in Turkey [12]. Improving safety culture is cited as a key action to reduce the risk of incidents in the care home setting [8]. Safety culture shapes the way report handlers process reports and use the reporting systems. Therefore, studying handlers' perceptions is essential for understanding and improving the effectiveness of incident reporting systems.

Studies have shown that the reasons for underreporting patient safety incidents are often related to the report processing process [11, 13]. These reasons include fear of consequences and blame, a lack of trust in anonymous reporting and a lack of feedback and actions after reporting [11]. More than 60% of university hospital health professionals reported that incident reporting may lead to disciplinary action [13], and one-fifth of software users did not trust the anonymity of submitted reports [6]. In addition, multiple other factors unrelated to the report processing phase can lead to the underreporting of incidents, such as a lack of time, reporting forms that are too long, direct discussion of the incident with persons involved and a lack of motivation to report others' mistakes [11].

To our knowledge, this is the first study to collect and describe handlers' perceptions of the report processing features of incident reporting software in the social and healthcare context. Therefore, this study offers novel insight into an understudied topic. The viewpoint used in this study is crucial, particularly in the context of developing future incident reporting. Incident reporting and reporting software in Finland will be reformed in the coming years, in line with the current Client and Patient Safety Strategy and Implementation Plan [14]. Thus, this study's topic is timely and can offer valuable knowledge to support the planned reform in Finland and future international development work.

Based on the identified research gaps and the ongoing reform in Finland, this study was conducted to address the following aims: (1) to identify the handling features of patient safety incident reporting software that support and challenge incident report processing; (2) to determine which handling features report handlers think should be added to current reporting software and (3) to describe shortcomings in reporting processes from reporters' perspectives.

## 2. Material and Methods

### 2.1. Design

This cross-sectional study administered the online survey “The Users' Perceptions of the Patient Safety Incident Reporting Software” [6], focussing on the qualitative analysis of its open-ended questions. A qualitative descriptive design was applied, which is suitable for obtaining direct and factual descriptions of the topic [15]. A qualitative content analysis [16] with quantification was used. SRQR reporting guidelines were followed [17].

### 2.2. Study Setting and Recruitment

The study was conducted in 2 of Finland's 21 wellbeing service counties, covering approximately 27,700 employees who are potential users of patient safety incident reporting software. Wellbeing service counties are responsible for organising public social and healthcare services. Public social and healthcare services encompass primary healthcare, specialised healthcare, oral healthcare, mental health and substance abuse services, services for people with disabilities and services for children, families and older adults, as well as social welfare [18]. The selected counties were chosen due to their large size and because they used two different electronic patient safety incident reporting software programmes, HaiPro [19] and Laatuportti [20]. Both programs are the most widely used incident reporting systems in Finland, and both feature similar reporting forms.

Incident reporters can be any employees in the counties, while report handlers are typically unit managers or persons in other chief positions. Unit managers or clinical teachers can utilise reporting software for other purposes, such as generating statistics or facilitating teaching. All employees were recruited from all units of the wellbeing service counties. The cover letter, the link to the UPPSIRS questionnaire and the link to a video presenting the research content, as presented by the first author, were emailed to unit managers by the organisations' contact persons, who were not members of the research group. Unit managers were asked to forward the email within their units, though not all may have done so. Another target organisation also shared the questionnaire link on their intranet.

### 2.3. Inclusion and Exclusion Criteria

All employees in wellbeing service counties, including healthcare professionals, social services staff, secretaries, experts and managers, can report patient safety incidents using electronic reporting software. Therefore, all potential software users, regardless of their job title or contract type, with experience using the software were eligible to participate in the survey. Health professional status was not required; for example, secretaries are active users and were therefore included. In the background questions, respondents were asked about their experience using reporting software. If a respondent indicated no experience with, the survey closed after these questions. Respondents who completed the survey were reporters of incidents, handlers or individuals who held both roles. Some users employed the software for purposes beyond reporting and handling reports, such as monitoring reports.

Nonemployee users, clients, patients and their family members were excluded. The questionnaire was available only in Finnish, requiring fluent Finnish skills.

### 2.4. Data Collection

Data were collected in January–February 2024 using the UPPSIRS survey developed for this study [6]. The online questionnaire was administered via Webropol, which is accessible from computers or mobile devices [21]. Unit managers were asked to forward the email and two reminders to employees in their units, without assessing whether employees should participate or not. In another target organisation, employees had access to the questionnaire on their intranet. The number of employees who received or noticed the research invitation is unknown. Therefore, a completion rate was calculated as follows: the number of completed questionnaires divided by the number of questionnaires opened by respondents, multiplied by 100 [22]. The questionnaire was open for a period of 4 weeks.

### 2.5. Data Analysis

This study analysed responses to four open-ended questions from the UPPSIRS survey, focussing on users' perspectives on patient safety incident report processing. Inductive content analysis was used [16] with quantification. Quantification provided an overview of the distribution and emphasis of categories within the data. Quantification did not aim to produce statistical information, but rather to deepen the understanding of the phenomena and offer additional insights that complement the qualitative interpretation. This design was suitable for analysing users' perceptions of incident reporting software because it allowed an in-depth exploration of individuals' experiences, and it emphasised nuances in the experiences that are difficult to achieve with purely quantitative approaches.

Analysis was conducted manually using Microsoft Excel. The first author (PhD student, RN) thoroughly reviewed all responses (*n* = 529) to the open-ended questions. Then, the meaning units (parts of the text) were extracted from four open-ended questions. The open-ended questions were (1) Which characteristics of the electronic system for reporting patient safety incidents support the reporting, management and analysis of incidents?; (2) Which factors prevent the electronic system for reporting patient safety incidents from fulfilling its task of reporting, managing and analysing incidents?; (3) Which features would you like to be changed or added to the electronic system for reporting patient safety incidents in the future? and (4) Please give any other comments on the topic or the questionnaire used. These questions were chosen for analysis because their responses reflected the participants' opinions on topics that aligned with the study's research focus. The meaning units were labelled with code, grouped into subcategories and then combined into categories based on content similarity.

The second (PhD, RN) and last author (PhD, RN) reviewed the analysis and suggested corrections. None of the researchers had done anything other than research-related work in the target organisations. The original data and the analysis were in Finnish. The first author, the native Finnish speaker, translated the final data analysis results into English. To ensure accuracy and clarity, two coauthors, also native Finnish speakers and fluent in English, independently reviewed and corrected the translation. An example of the data analysis is shown in Table 1, with respondents' codes in square brackets. The total number of meaning units in each category was counted using quantification. Respondents' characteristics were presented using frequencies and percentages.

### 2.6. Ethical Considerations

Participation was voluntary, and participants could discontinue at any time. The research invitation, privacy statement and study announcement were sent to all potential participants. At the beginning of the questionnaire, informed consent was obtained by clicking the “next” button, which was accompanied by the following bolded statement at the end of the first page of the questionnaire: “By clicking the 'next' button, I confirm my voluntary participation in this study, accepting and confirming the above information.” Those unwilling to participate were guided to close the browser window by clicking the close button.

The questionnaire was conducted using Webropol, a secure platform that complies with the General Data Protection Regulation [21]. No direct identification data were collected; the indirect personal data were reported in grouped form (Table 2). Only the first author accessed the data with indirect identifiers, stored on a BitLocker-secured memory stick, and kept in a locked drawer in the first author's office. The results are reported on a group level. The organisations granted permission for this study. The Finnish National Board on Research Integrity TENK guidelines [23] were followed, which did not require an ethical statement to be applied. This was confirmed by the Research Ethics Committee of the University of Eastern Finland (No 17/2023).

## 3. Results

### 3.1. Characteristics of the Data

A total of 1398 potential users of patient safety incident reporting software opened the online survey, and 755 responded to it. Therefore, the completion rate was 54%. Of the respondents, 117 shared their perceptions of this study's interests in the free-text portion of the UPPSIRS survey. Most respondents were incident reporters (*n* = 52, 44%) or reporters and handlers (*n* = 45, 39%; Table 2). The average age of the respondents was 47 years, and most were female (*n* = 102, 87%). Most respondents (*n* = 96, 82%) had over 10 years of work experience in social work and/or healthcare and a background in nursing (*n* = 77, 66%). Managers were 42% (*n* = 49) of the respondents. The respondents worked in various units across the wellbeing service counties. Most respondents (*n* = 42, 36%) worked in social services such as home care and disability services. Other respondents worked in specialised healthcare (*n* = 34, 29%), primary healthcare (*n* = 28, 24%), administration (*n* = 8, 7%) and other services (*n* = 5, 4%).

### 3.2. Features Supporting Incident Report Processing

Of incident report handlers, 26 shared their views on the software features that facilitate incident report processing. Suggestions were coded (*n* = 34) and combined into 15 subcategories and 6 categories. The supporting features were transmission to handlers and units (*n* = 10); easy and straightforward reporting software (*n* = 7); management of reports (*n* = 5); reports and graphics (*n* = 5); design and structure (*n* = 4) and analysis tools (*n* = 3).

Respondents were satisfied with the incident reports' *transmission to handlers and units*. Reports were transmitted to the correct unit automatically, and they immediately received an automated email notifying them that the reports had arrived.

Automatic notification (is given) to the handler when the report has been made, and this message already briefly states the content of the incident report. [468]

*Easy and straightforward* report processing in the software was seen as a supporting feature in practice. Some respondents' opinions also reflected that the *management of the reports* functioned well. Unprocessed reports stood out from the list of reports, and the software sent reminders about them: *“Unprocessed reports can be easily found in the list.”* [454]. The processing phase of each report was easily accessible through the software. Another useful software feature enabled the transfer of reports to another unit and the removal of null reports, which was sometimes necessary.

The participants considered *the reports and graphics* that handlers could obtain from the software to be useful. Using a reporting tool, they could procure a comparison report and compare different years. The software's *design and structure* supported the processing of the reports. The reporting form's fields were colour-coded and included a place to define a person in charge of the measures: *“The colour codes in the answer sheet support reporting and processing.”* [475] The structure of the form helped illustrate the primary cause of the incident.

Furthermore, the respondents reported that the software's *analysis tools*, such as the root analysis tool and risk evaluation table, worked well: *“A root cause analysis is a good tool for* processing *and analysing the reports.”* [450]

### 3.3. Features Challenging Incident Report Processing

Features that participants found challenging in incident report processing were coded (*n* = 43) and grouped into 19 subcategories and 7 categories. The challenging features were as follows: poor technical functionality (*n* = 15); a lack of suitable classification and answer options (*n* = 7); report transmission from a reporter to a handler and units (*n* = 7); too many fields (*n* = 5); inadequate reporting features for handlers (*n* = 4); challenges in forwarding reports (*n* = 3) and anonymous reporting (*n* = 2).

The software's *poor technical functionality* caused challenges in reporting processing. The software generally worked slowly, especially when saving and updating. Respondents also stated that specific parts of the software were time-consuming, such as the root cause tool and numerous drop-down menus. The respondents described the software as complex and laborious to use. Sometimes, the software crashed during report processing, and the process had to be restarted. Printing from the software was also difficult.

The software is terribly SLOW from the handler's perspective—processing takes an unreasonable amount of time due to the clumsiness and slowness of the software, and because of this, reports must be printed and recorded into the software after processing. [421]

Respondents identified *a lack of suitable classification and answer options* in the software. Predefined answer options were not always suitable for reports. Respondents reported that finding suitable classifications for their represented units was sometimes challenging. For example, the incident severity classification was not entirely suitable for laboratory services. Not all units and partners were listed in the software; therefore, transferring the report to the correct unit was not always possible. The software also limited the possibility of reporting all medicines related to the incidents in a structured way. Only two medicines could be reported in a structured manner; however, more than two medicines were sometimes involved in a single incident. A lack of free-text fields limited processing when a handler was forced to use predefined answer options:

The software limits the amount of information that can be entered there, e.g. in reports related to medicines, you can structurally enter a maximum of two medicines, even if it is often necessary to enter several - the total information obtained remains incomplete. [522]

The respondents also reported challenges in *transmitting reports from reporters to handlers and units*. Some respondents described how reports frequently ended up in the wrong units. Reports needed to be transferred from one handler to another (correct) handler. If a report was transferred to the wrong handler, the correct handler could not see the report before the initial recipient transferred it. Furthermore, staff changes were not updated in the software, which caused problems in report transmission. If an external service provider was involved in the incident, the handler needed to inform them about it in other ways, as external providers did not use the software, and it was not possible to forward reports to nonusers:

Information is directed to the wrong person, in which case the correct person does not even see that the report has been made. [47]

The software required *too many fields* to be filled out during report processing. Some fields required responses that were too detailed. From some respondents' perspectives, the software included unnecessary fields:

The current one is too difficult for a handler. A new field always opens, and you can never be sure if you have finished processing until the end. [463]

*Inadequate reporting features for handlers* meant that statistics could not be filtered in all the necessary ways, and the extent of the available reports was not broad enough. Because the software did not offer some reporting features, the needs of the handlers and organisation were not met. Inadequate features forced handlers to complete some reports manually:

The software does not produce reasonable summaries that can be used in monitoring, and the monitoring/statistical work has to be done manually. [520]

*Challenges in forwarding reports* were related to two issues. First, identifying the correct unit to forward reports to was challenging. This was because the list of potential units included all the wellbeing services that the county's units offered and was thus very long. Second, when the report was forwarded to the correct handler, the report remained visible to the initial recipient. This was unnecessary in cases in which the initial recipient was the wrong handler for the report in question. Third, forwarding to another operating area's upper management (e.g., from acute care to home services) was not possible:

…the report cannot be transferred to upper management during the processing phase other than management in your own ‘service line', e.g. from wards to the supervisors of the wards' operating areas, even though I know that the incident happened in the part of the health and social services centre and information about the incident should be given to them. [493]

Some respondents found *anonymous reporting* challenging. The software did not require information on the reporter or the client/patient to be reported. Some respondents found the lack of client information challenging from the report processing perspective:

If the resident was immediately recognisable from the report, intervening would be faster. It takes time to find out who the resident is so that you can fix the issue. [483]

### 3.4. Necessary Improvements to Handling Features in Reporting Software

Respondents suggested several handling features that should be added to the current reporting software in the future. Suggestions were coded (*n* = 32) and combined into 19 subcategories and 8 categories. The necessary new features were as follows: technical handiness (*n* = 11); report visibility (*n* = 4); changes to the anonymity of reporting (*n* = 4); broader classification and answer options (*n* = 4); support for the planning of measures (*n* = 3); improved statistical tools (*n* = 3); reminders of unprocessed reports (*n* = 2); and improved transparency of report processing (*n* = 2).

Respondents expressed the need for improvements in *technical handiness*. The drop-down menu could include search options, and the background units of handlers and reporters could be automatically populated in the report. The software could utilise links, such as links from the incident type to a summary of all similar incident types and from the announcement of the new report straight to report processing: *“There could be a link to the statistics—how many similar/related incidents have been made,* for example, *by then or compared to the previous year”* [494]. Furthermore, the respondents expressed that it should be possible to separate reports only related to nurses or physicians. Generally, the respondents wanted faster and more accessible software.

Respondents suggested matters related to *report visibility*. They proposed that handlers should be able to see all reports related to the unit, as this would solve a lag in report processing if the initial handler were absent. Currently, handlers do not know who else can see the report, and thus, discussing who should start the report processing is impossible. Therefore, handlers should be able to see who else can view the report. When the report concerns two different units, it should be transmitted to both units and processed in cooperation between them. It should be possible to forward the reports to upper management:

You should be able to see the incidents of the entire unit and not just the locations you are responsible for. For example, you cannot handle the incidents if a colleague is away for a long time or on vacation. [439]

Respondents stated that they would make *changes to the anonymity of reporting*. Some respondents suggested that handlers should have the right to access the reporter's information, even if the report were anonymous. Another suggestion was that handlers should be able to call the reporter if necessary. During a phone call, the reporter and the handler could supplement the report using protected remote access. In addition, respondents expressed that the client's information should be visible to the handler:

In housing services for the elderly, it would be important to see who the resident is from the software. It would be much easier to think about resident-based measures and means and what should be changed. [419]

*Broader classification and answer options* were suggested. This could enable the processing of different patient safety incident types more comprehensively: *“The features should be expanded to support processing several types of patient safety incidents”* [470]. In future, the software should consider all types of social and healthcare units in its classifications. Limitations, such as the number of medicines that can be reported in a structured way, should be addressed.

The respondents stated that the software should *support the planning of measures* if reporters make their suggestions for the incident's solution. Structured action proposals for recurrent incidents could be provided in the software. In addition, the respondents expressed that the software should force the handler to write their action proposal: *“ In processing reports, there should be a forced function that every report must include measures”* [529].

Respondents suggested that the software should send *reminders of unprocessed reports*. Reminders could be sent to the handler's email after specific intervals. *Improved transparency of report processing and improved statistical tools* were also suggested: *“A better statistical tool that would enable better comparison”* [529].

**Figure 1:**
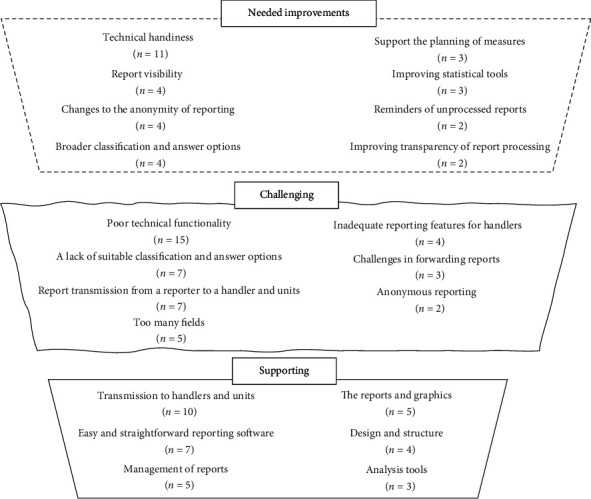
Summary of the features supporting and challenging incident report processing and necessary improvements based on the qualitative inductive content analysis of respondents' (*n* = 65) perceptions. The total number of meaning units in each category is mentioned in brackets.

A summary of the findings (features supporting and challenging incident report processing and necessary improvements) is presented in Figure 1.

### 3.5. Shortcomings in Report Processing

Of the incident reporters, 52 respondents reported shortcomings in reporting processes. A total of 65 meaning units were identified and categorised into 18 subcategories and 7 categories (Table 3). Shortcomings in the report processing were as follows: incident reporting rarely leads to concrete changes or actions (*n* = 19); challenges in shared learning (*n* = 14); reporters do not receive feedback or information about processing or measures (*n* = 12); blame or fear of consequences (*n* = 7); processing times are long (*n* = 6); the underestimation of the reports (*n* = 4) and report processing is not objective and depends on the handler (*n* = 3).

Among respondents, a common opinion was that *incident reporting rarely leads to concrete changes or actions*: *“Even if you make a report, it will not affect your work, and the issue will not change”* [304]. Respondents suspected that reports were not analysed and processed, or mentioned that report processing was unclear for them. A discussion was not considered adequate if it was the only action taken after an incident was reported.

*Challenges in shared learning* reflected the issue that reports were not discussed together. Respondents reported that incident reports were not discussed with work units, staff, team or a reporter: *“They* (incident reports) *have not been discussed at all with the staff for at least two years”* [281]. Additionally, processing was restricted; reports were discussed at most once in a ward meeting, and their further processing was often inadequate. Report processing as a group was seen as necessary for incident reporting to produce real benefits.

The respondents stated that *reporters do not receive feedback or information about processing and measures*. Respondents were not happy with the automatic “thank you” message sent by the software. It was not possible to follow the process in real time, and reporters were unaware of who handled the reports they submitted. Information that someone had read the report was unavailable, as were the final measures after processing:

After making the report, you usually do not receive any information as to whether it has been processed and whether the report leads to any measures or development proposals. [142]

Report processing could lead to *blame and fear of consequences*. A manager or other unit manager could blame a reporter, pressure them to change a report or report less information in future. Report processing often involved blaming or perceived blaming of staff. Respondents described fear of consequences, such as managerial actions after reporting:

Another unit manager's reply and instruction to change the content (of the report) felt oppressive. Kind of, I, as a reporter, was somehow accused at that point. [371]

The respondents indicated that the *processing times* for incident reports *are long*. Processing is slow and can take a considerable amount of time, especially if a unit manager is absent. Respondents also described *the underestimation of the reports*. The risks and fears of employees were not taken into account. Respondents perceived that incident reports were not appreciated: *“Incidents and risks are often downplayed*---*”* [388].

The respondents expressed that *report processing is not objective and depends on the handler*, as a lack of external opinion and inadequate training of handlers can be observed in report processing. Methods of processing varied depending on unit managers and team managers. Furthermore, the respondents indicated that processing had changed; earlier, more than one person processed and analysed the reports, which was considered a better procedure than the current one:

Also, reports used to go to several people for processing and analysis. Now, it's up to the department head, staff nurse, and chief physician to run them. The processing is not objective. [231]

## 4. Discussion

This descriptive study examined patient safety incident reporting software from the perspectives of report handlers and reporters. The study identified software features that support and challenge incident report processing and highlighted development needs. Additionally, shortcomings in report processing from the reporters' perspectives were described. While previous studies have identified barriers to incident reporting [2, 11], this study highlighted shortcomings in incident reporting systems.

Although respondents identified features of incident reporting software that supported their work as report handlers, the number of challenging features was higher. The improvement was the technical functionality of the software. In this study, handlers described how they manually compile statistics and reports or print incident reports to fill them out due to the poor technical features and slowness of the software. Incident reporting software can facilitate the processing of reports and make it more effective. However, current systems do not utilise all the possibilities this era of technologies enables. The number of incident reports is significant [2, 4], and report handlers are often unit managers with multiple other duties in addition to incident report processing.

This study includes detailed descriptions of challenges and development needs regarding software's handling features. The solutions to these problems could be found through artificial intelligence, as its potential in improving patient safety has already been identified [24]. Front-line workers may not even recognise all the possibilities artificial intelligence offers for report processing. For example, factors that assist in mitigating patient harm after an incident have occurred were not related to incident reporting software in a large systematic review of the home care setting [8]. Real-time analysis of reports with artificial intelligence could potentially mitigate patient harm.

This study strengthens the findings of previous studies from a different perspective. The need for more precise classification in incident reporting software has been recognised in previous studies from reporters' perspectives [3, 6]. According to the results of this study, a lack of suitable classification is identified from the report handlers' perspectives. Unsuitable classification can lead to distorted reporting statistics, and some incidents might be hidden under insufficient categories. On the other hand, in this study, the respondents identified some software features that support report processing; for example, the root cause analysis tool is used to reduce risk in many countries as part of the report processing [8].

This study revealed that typical barriers to incident reporting remain unaddressed. Incident reporters stated that reports were not discussed within workplaces; employees could not see any concrete changes resulting from reporting; reporters did not receive any feedback about their reports after submitting them; discussions around reports often led to blaming; reported incidents were underestimated and report processing was not objective. Poor report processing remains a significant barrier to the effective utilisation of incident reporting software [2]. Several reasons for underreporting are related to report processing, including a lack of feedback, visible actions and fear of blame and consequences [11]. These shortcomings in report processing were also identified in this study. The difference to previous studies [11, 25] was that in this study, reporters stated that the underestimation of the reported incident was one of the shortcomings in report processing.

Although incident reports are expected to lead to visible actions [2], previous research [4] and this study have revealed that actions do not always occur. In this study, reporters' responses conveyed frustration with the lack of concrete actions taken after their reports. Furthermore, reporters often experience a lack of feedback, which can reduce their motivation to report [11]. This study showed that automated feedback is not the solution when reporters expect detailed information about report processing rather than general automated messages. Shared discussion and processing of reports within units can enable employees to be more involved in the report processing process. A previous study found that higher involvement is associated with an increased number of incident reports [26]. Thus, the results emphasise the importance of timely visibility into incident report processing for reporters.

This study shows that the handling features of patient safety incident reporting software require improvement. In addition, this study identified shortcomings in incident report processing that must be solved as part of the units' safety culture and as part of software development in future. This study highlights the importance of involving users in the development of healthcare technologies.

### 4.1. Strengths and Limitations

One of the strengths of this study is related to the survey used. The UPPSIRS survey was carefully developed following DeVellis's tool development steps [27], and its development is described in detail elsewhere [28]. Data were collected from two wellbeing service counties using the two most widely adopted electronic incident reporting software programs in Finland. The study included all social and healthcare units, including public social, primary and specialised healthcare services.

Most of the respondents had a nursing background, which is a limitation considering that the study's focus was on software users' perceptions of the topic in general. Nurses are the most common reporters and report handlers, who explains this distribution.

The credibility was enhanced through a careful review of the original data and a meticulous analysis process, in which two research group members independently checked the categories created by the first author and suggested corrections. Researchers' professional background was disclosed. Transparent reporting of the study context, analysis, and findings, along with delayed public data sharing, supports confirmability. Transferability of the results should be considered primarily within comparable healthcare contexts, where organisational structures and professional roles are similar. Detailed documentation of the entire research process, along with a clear description of the study's objective, methods and data collection, ensures dependability [29].

This study has several limitations. In one organisation, employees were able to participate only if their unit manager forwarded the research invitation to them. This may have caused selection bias. Most respondents had a nursing background, as expected. This may have biased the findings, as results primarily reflected nurses' perceptions. It is possible that perceptions among other occupational groups would have been different, as it is known that reporting habits vary across occupational groups.

Although incident reporters broadly described shortcomings in report processing, it is worth noting that the UPPSIRS survey did not include questions specifically targeting this topic. The survey focused on the incident reporting software and its features, and some participants may have limited their responses accordingly. We decided to analyse respondents' perceptions of report processing because issues were reported in the responses that were considered relevant to the study's goals. Respondents' perceptions included valuable information from incident reporters' perceptions of shortcomings in report processing.

The data collection setting included two different incident reporting software. Although the content of the reporting forms in the software was almost identical, it is possible that the research group was not familiar with some differences between the software. These differences were not the primary focus of this study; however, they may have influenced participants' perceptions and should be considered when interpreting the results.

### 4.2. Implications for Nursing Management

Incidents reported from all occupational groups are valuable and can prevent future harm [5]. Professionals with nursing backgrounds play a key role in incident reporting and report processing [7, 8, 30], with most report handlers being unit nurse managers.

Failure to consider the practical knowledge of software users about challenges in reporting software might cause underreporting, nonreporting, and suboptimal utilisation of the reports. This study emphasises the importance of incorporating end users into incident reporting software development processes. Nurses' perceptions are especially crucial, as they represent the largest user group. Nurse managers must be able to participate in all phases of software development, not just software testing, when discussing software handling features. Software handling features must support, not challenge, incident report processing.

Report processing should promote shared learning. This study demonstrates that the report processing structures and methods require improvement and reform. In addition to technical and content reform planned for the current reporting software in Finland [14], standard practices around incident reporting software must be implemented in units and organisations to utilise the valuable reported information.

## 5. Conclusions

The findings indicate that it is time to critically evaluate the reporting software used, as report handlers were dissatisfied with multiple software features for report processing. To ensure the optimal software for unit managers and incident report handlers to process valuable information on client and patient safety, they must be involved in the development processes of those systems. Furthermore, incident report processing procedures require changes to ensure that reporters feel that it is meaningful and secure to report all patient safety incidents they observe or are involved in. Standard report processing procedures are necessary because the current report processing is biased from the reporters' perspectives. Reporting software with advanced features could solve issues with the software features recognised in this study.

## Figures and Tables

**Table 1:** An example of the data analysis.

Theme	Category	Subcategory	Code	Meaning unit [000] = the code of the respondent
Shortcomings in the report handling processes	Blaming and fear of consequences	Fear of consequences from managers	Reporters are afraid of consequences.	*“Some are also afraid of consequences from the person in charge.”* [394]
		The processing of reports is perceived as blaming	Processing reports is challenging because it is perceived as blaming.	*“In many work communities, report handling is still challenging, the matter is (sensitively) perceived as blaming or reprimanding.”* [388]
		Processing of reports with blame	Managers often blame staff.	*“The assumption is that going through the HaiPro* (reports) *in the ward meeting is enough, and even then,* (staff are) *often blamed.”* [231]
			Managers aim to blame those involved.	*“…they aim to blame those involved.”* [203]
			Blame is placed for what happened.	*“Or blame them about them* (incidents)*…”* [154]
		Blaming of reporters	A manager pointed out and questioned a reporter who made many reports.	*“Previously, I made many HaiPro* (incident) *reports, which led to reprimanding where the unit manager asked, what is wrong with me when so many incidents happen to me/I observe so many incidents.”* [469]
			Another unit's manager asked to change a report and made a reporter feel guilty.	*“Another unit's manager contacted me and instructed me to change the content of the report. I felt distressed. As a person who had submitted the report, I felt somewhat accused at that point.”* [371]

**Table 2:** Characteristics of respondents (*n* = 117).

Respondents' characteristics	*f*	%
*Patient safety incident*		
Reporter	52	44
Reporter and handler	45	39
Handler	14	12
Another user (such as a quality manager or teacher)	6	5

*Gender*		
Female	102	87
Male	12	10
I prefer not to say	3	3

*Age*		
18–34	20	17
35–59	81	69
Over 60	16	14

*Work experience in social services or healthcare*		
Under 5	7	6
5–10	14	12
Over 10	96	82

*Working area*		
Social services (disability services, home care, residential services for older people, social welfare services, etc.)	42	36
Specialised healthcare (intensive care unit or high-dependency unit, inpatient ward, outpatient clinic, out-of-hours services, etc.)	34	29
Primary healthcare (health centre, health centre's inpatient ward, oral healthcare, rehabilitation or special services, etc.)	28	24
Administration	8	7
Other services (laboratory service, imaging service, support and rescue service unit)	5	4

*Current work job title*		
Nurse manager or social care manager	37	32
Registered nurse, public health nurse, paramedic or clinical nurse specialist	30	26
Practical nurse, nursing assistant or dental assistant	12	10
Social worker, social counsellor and assistant in social services (e.g., disability counsellor)	10	8
Others (e.g., clinical nursing or medicine educator, pharmacist, project worker, psychologist, rehabilitation or special worker and secretary)	10	8
Physician manager	7	6
Manager (not specified whose)	5	4
Expert (e.g., patient safety expert)	3	3
Physician or dentist	3	3

A total	117	100

**Table 3:** Shortcomings in report processing from reporters' (*n* = 52) perspectives.

Category	Meaning units
Incident reporting rarely leads to concrete changes or actions	19
Challenges in shared learning	14
A reporter does not receive feedback or information about the processing or measures	12
Blame or fear of consequences	7
Processing times are long	6
The underestimation of the reports	4
Report processing is not objective and depends on the handler	3
A total	65

*Note:* The total number of meaning units in each category is mentioned in the last column.

## Data Availability

The research data are permanently archived without personal data at the Finnish Social Science Data Archive. After January 1, 2026, the archive may hand over the data for reuse to registered clients for research, teaching and study.

## Supporting Information

Supporting 1: Standards for Reporting Qualitative Research (SRQR).
